# Brief Online Negative Affect Focused Functional Imagery Training Improves 2-Week Drinking Outcomes in Hazardous Student Drinkers: a Pilot Randomised Controlled Trial

**DOI:** 10.1007/s12529-021-10019-9

**Published:** 2021-08-25

**Authors:** Ruichong Shuai, Alexandra Elissavet Bakou, Jackie Andrade, Leanne Hides, Lee Hogarth

**Affiliations:** 1 School of Psychology, University of Exeter, Washington Singer Building, Perry Road, Exeter, EX4 4QG UK; 2 School of Psychology, University of Plymouth, Plymouth, UK; 3 School of Psychology, University of Queensland, Brisbane, Australia

**Keywords:** Pilot randomised controlled trial, Emotion regulation, Guided imagery, Negative affect drinking

## Abstract

**Background:**

Negative affect plays an important role in motivating problematic alcohol use. Consequently, training imagery-based adaptive responses to negative affect could reduce problematic alcohol use. The current study tested whether personalised online functional imagery training (FIT) to utilise positive mental imagery in response to negative affect would improve drinking outcomes in hazardous negative affect drinking students.

**Method:**

Participants were 52 hazardous student drinkers who drink to cope with negative affect. Participants in the active group (*n* = 24) were trained online over 2 weeks to respond to personalised negative drinking triggers by retrieving a personalised adaptive strategy they might use to mitigate negative affect, whereas participants in the control group (*n* = 28) received standard risk information about binge drinking at university. Measures of daily drinking quantity, drinking motives, self-efficacy and use of protective behavioural strategies were obtained at baseline and 2 weeks follow-up.

**Results:**

There were three significant interactions between group and time in a per-protocol analysis: the active intervention group showed increased self-efficacy of control over negative affect drinking and control over alcohol consumption and decreased social drinking motives from baseline to 2-week follow-up, relative to the control intervention group. There were no effects on drinking frequency.

**Conclusion:**

These findings provide initial evidence that online training to respond to negative affect drinking triggers by retrieving mental imagery of adaptive strategies can improve drinking-related outcomes in hazardous, student, negative affect drinkers. The findings support the utility of FIT interventions for substance use.

## Introduction

Hazardous drinking is prevalent within the UK undergraduate population (defined as greater than 8 on the Alcohol Use Disorders Identification Test) [1, 2]. Although students drink for a variety of reasons [3], drinking to cope with negative affect is a key prospective risk factor for binge drinking [4] and alcohol dependence [5]. Furthermore, drinking to cope with negative affect mediates hazardous drinking in college students who have experienced psychiatric symptoms or trauma [6–8]. Consequently, many therapeutic approaches seek to mitigate drinking to cope with negative affect in order to reduce alcohol problems. For example, mindfulness-based interventions which train individuals to identify and mindfully accept negative affect have been shown to attenuate the effect of stress induction on emotional state [9–12] and alcohol-seeking behaviour [13], reduce alcohol craving and relapse [14–17] and promote long-term abstinence relative to treatment as usual [18]. These findings support the utility of targeting negative affect–induced alcohol seeking as a therapeutic strategy for substance use [19].

Imagery-based interventions have been similarly shown to improve emotional and substance use outcomes. Specifically, training individuals to imagine their best possible future self in response to negative affect has been found to improve symptoms of depression and anxiety [20, 21]. Similarly, episodic positive future thinking studies have found that instructing participants to think of achieving positive future goals improves mood [22], reduces alcohol demand in college students [23] and alcohol-dependent individuals [24], reduces alcohol consumption and increases the use of protective behaviour strategies (to reduce problematic drinking) in heavy drinking college students [25]. Episodic positive future thinking also reduces food intake [26, 27] and promotes weight loss in overweight individuals [28]. However, only one study has shown that episodic positive future thinking can reduce alcohol consumption in field testing (i.e. outside the lab) [25], indicating greater need for evaluation of this approach.

Functional imagery training (FIT) is another imagery-based intervention designed to promote health behaviour change. The unique focus of FIT is to train individuals to build an imagery retrieval routine into daily life, whereby positive mental imagery about the positive outcomes resulting from behaviour change is evoked systematically over time. For example, in several studies, overweight participants were trained to practice retrieving mental imagery of how they will work towards their healthy eating goals and the positive impact that will follow if they achieve these goals (e.g., feeling good about themselves), at regular times during the day such as when brushing teeth or walking upstairs, as well as when actually craving food [29, 30]. The theoretical objective is to link risk situations with the retrieval of alternative goal images, to promote behaviour change (i.e. increase abstinence). This therapeutic approach has been shown to reduce snacking [29] and maintain weight loss over time [30]. Importantly, no FIT study has examined therapeutic effects on substance use outcomes. Therapeutic approaches akin to FIT have been shown to improve emotional self-regulation, reduce self-harming [31] and increase grit, the ability to persevere with work towards a goal in the face of challenges [32]. Although these foregoing studies suggest that a FIT intervention focused on reducing reactivity to negative affect might have efficacy in attenuating negative affect motivated drinking behaviour [33], this prediction has not been tested directly.

Most of the aforementioned studies have employed in-person interventions. Electronic administration can widen access, facilitate extended training and embed training within the client’s natural environment, but fewer studies have tested the efficacy of the electronic delivery format. A small number of episodic positive future thinking studies with electronic delivery have shown effects on demand for food and substances [28, 34, 35]. Only one FIT study used a mixture of face to face and electronic communications and demonstrated improvement in weight loss [30]. Consequently, it remains to be seen whether an entirely electronically delivered FIT protocol would improve substance use outcomes, supporting the efficacy of this format.

The purpose of the present study was to test whether a brief online negative affect–focused FIT intervention would improve drinking outcomes in hazardous student drinkers, who reported drinking to cope with negative affect. Participants receiving active intervention were initially trained to respond to personalised negative affect drinking triggers by retrieving a mental image of themselves employing an adaptive strategy to mitigate negative affect. The objective was to replace drinking to cope as the established cognitive response to negative affect, with a mental representation of an adaptive strategy, thus breaking the link between negative affect and drinking [36]. The control group received standard university binge drinking health information. All participants received emails/text messages across the 2-week follow-up period to promote and quantify engagement with the intervention [37]. Drinking outcome measures were recorded at baseline and 2-week follow-up and included daily drinking quantity and questionnaires assessing the self-efficacy of control over drinking especially in the context of negative affect, motivation to drink in different scenarios, and the use of protective behavioural strategies to limit drinking (pre-registered protocol on As Predicted #20,375: https://aspredicted.org/see_one.php). The active intervention group was expected to show improvements in all drinking outcome measures. However, because reductions in drinking quantity are generally not seen in short timeframes, it is more likely that intervention effects will be observed on drinking-related outcomes such as self-efficacy of control over negative affect drinking and related measures [3, 38].

## Methods

### Participants

**Fig. 1:**
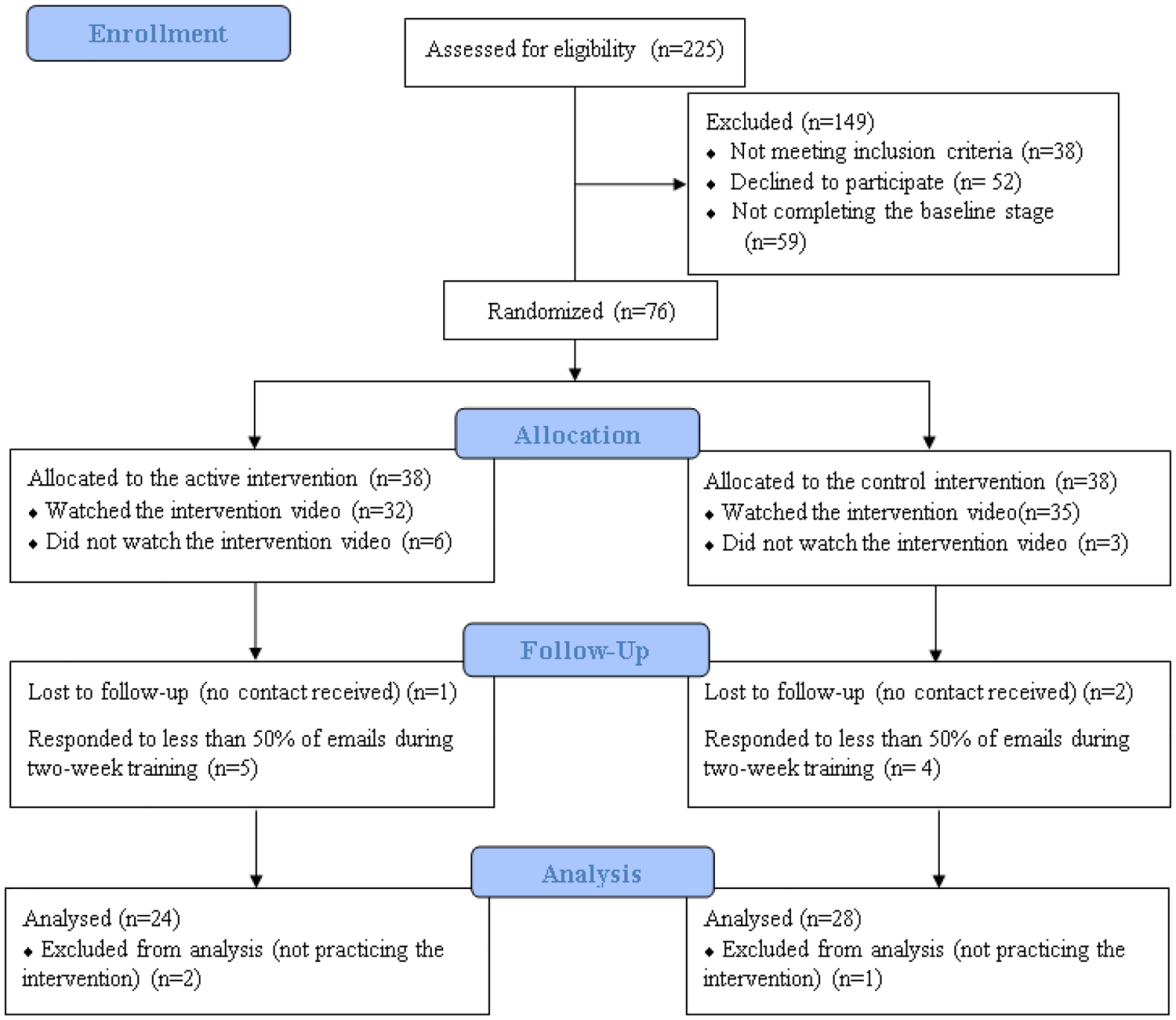
Consort diagram displaying the progress and attrition through the intervention and follow

Participants were recruited using convenience sampling from the Exeter psychology research participation system Sona website and Facebook page ‘Overheard at Exeter’ and screened in the study. Of 225 initially screened, 52 participants completed the whole study and were included in the final analysis (see screening measures below and Fig. 1). The target sample size after exclusion was sought to be close to previous proof-of-concept FIT trials (*N* = 24–45) [29, 31, 32].

### Materials

#### Screening Measures.

Screening was used to select hazardous student drinkers who drink to cope with negative affect. Screening questionnaires asked participants their age (only those aged 18–25 years were included), sex, and the frequency of current drinking (only those who reported to drink at least monthly were included). The 10-item Alcohol Use Disorder Identification Test (AUDIT) was completed to assess the frequency of alcohol use and alcohol-related negative consequences experienced in the past 12 months. Total scores range from 0 to 40, with scores ≥ 8 identified as hazardous drinking in college students [39] (only hazardous drinkers were included). The Drinking to Cope Checklist (DTCC) contains 35 negative affective states (items) which participants can endorse as triggering their alcohol drinking with ‘Yes’ or ‘No’ responses (e.g., ‘I am more likely to drink when I feel stressed’) [40]. In this study, half of the student sample endorsed ≥ 5 items, so this cut-off for inclusion criterion was chosen to select the top half of participants in terms of negative affect drinking.

#### Intervention.

The two intervention videos (active or control) were PowerPoint presentations containing text and images, with the text read out by the same female voice while it was presented on screen. The active intervention video was 8 minutes and contained 13 pages over which two main points were communicated (available in full at YouTube link: https://youtu.be/xbhTxFCcRjY). The opening summary statement was: ‘Your questionnaire responses indicated that you are at increased risk of alcohol dependence in the future. This is because you drink to cope with negative emotions, such as depression, stress and boredom’. (1) Participants were provided with a personalised list of the negative affective states that they had endorsed as motivating their drinking on the DTCC and were told that drinking to cope is a risk factor for dependence in the future, and that part of the training was to become aware of these triggers. (2) The training component was summarised by the statement: ‘This video will teach you a “reactive imagery” technique, to deal with negative emotions, which will help reduce your risk of alcohol dependence in the future. The reactive imagery technique is very simple: When you experience negative emotions (such as those you would drink to cope with), react by vividly imagining your best self — you on a good day — and ask “what would your best-self do now?” Then do those positive activities’. A guided imagery procedure was then followed which asked participants to vividly imagine their negative drinking triggers, and then their best self, and the adaptive strategy they could adopt to mitigate negative affect. Training ended by participants being told that they should practice this reactive imagery technique over the next 2 weeks.

The control intervention video was 4 minutes and contained 13 pages. The content summarised the binge drinking risk information derived from the US National Institute on Alcohol Abuse and Alcoholism (NIAAA) College Drinking Factsheet [41] (see also YouTube link for video: https://youtu.be/08ra9hBqcf4).

#### Baseline and Outcome Measures.

The same questionnaire scales were completed at baseline and follow-up. Questionnaires measured participants’ experience over the past 2 weeks (except for the ‘Self-efficacy’ scale, as detailed below), to reflect equivalent time span pre and post-intervention. All questionnaires have been previously validated in the undergraduates or young people aged 17–25 who abuse substances, which was similar to our selected sample. Cronbach’s α was calculated for scales, and most of them had good internal consistency (see Table 2 for details).

The Daily Drinking Questionnaire — Revised (hereafter ‘Daily Drinking’) was used to measure drink type and volume consumed in each of the past 14 days, rescored as sum of alcohol units consumed over the past 14 days [42].

Protective Behaviour Strategies Scale-Revised (hereafter ‘Protective Behaviours’) was used to assess the use of various strategies to control drinking behaviour in the past 2 weeks with 20 items (e.g. ‘Determine not to exceed a set number of drinks’), endorsed on a 6-point Likert scale ranging from 1 (‘Never’) to 6 (‘Always’), averaged to yield a single score [43].

Controlled Drinking Self-Efficacy Scale (hereafter ‘Self-efficacy’) contains 20 items, which assessed participants’ confidence in controlling drinking in the next 6 months for various risk scenarios, endorsed on a scale ranging from 0 (‘not confident’) to 10 (‘very confident’) [44, 45]. The scale contains 4 subscales which evaluate different risk scenarios: negative affect (‘when you are irritated’), frequency of drinking (‘Can you stop yourself from drinking alcohol at least one day a week’), positive mood/social context (‘when you want to feel more confident’) and consumption quantity (‘Can you make sure that you do not have more than three drinks on any time that you have a drink’). Higher score indicates an increased confidence in controlling drinking in the certain situation, e.g., higher score on negative affect subscale indicates that the respondent is more confident in controlling drinking when experiencing negative affect.

The Drinking Motives Questionnaire—Revised is a five-dimensional questionnaire measuring drinking motives in the past 2 weeks, which will hereafter be labelled as ‘Drink Motives-anxiety’ (e.g. ‘to relax’), ‘Drink Motives-depression’ (e.g., ‘to numb my pain’), ‘Drink Motives-enhancement’ (e.g. ‘to get a high’), ‘Drink Motives-conformity’ (e.g., ‘to be liked’) and ‘Drink Motives-social’ (e.g. ‘as a way to celebrate’), endorsed on a scale ranging from 0 (‘never’) to 10 (‘always’) [46]. Drink Motives-anxiety and Drink Motives-depression were highly correlated at baseline, *r* = 0.78, *p* < 0.001, and at follow-up, *r* = 0.84, *p* < 0.001, and so were averaged into one single ‘Drink Motives-coping’ score for analysis.

The Generalised Anxiety Disorder Questionnaire (hereafter ‘Anxiety’) containing 7 items was used to measure the symptoms of generalised anxiety disorder (e.g., ‘feeling nervous, anxious or on edge’) in the past 2 weeks. The score on each item ranges from 0 (‘Not at all’) to 3 (‘Nearly every day’). The total score can range from 0 to 21, with a score of 10 as the cut-off point for moderate anxiety [47]. This measure has been validated to measure anxiety in the general population [48] and showed an excellent internal consistency in an undergraduate sample [49].

The Patient Health Questionnaire Depression Scale (hereafter ‘Depression’) containing 8 items was used to measure symptoms of current depression (e.g., ‘little interest or pleasures in doing things’) in the past 2 weeks. The score on each item ranges from 0 (‘Not at all’) to 3 (‘Nearly every day’). The total score can range from 0 to 24, with a score of 10 as the cut-off point for moderate depression. This measure has been validated in the general population [50] and college students [51].

### Procedure

The study lasted for a 6-week period from the end of May to beginning of July 2019. The whole procedure was conducted by the first author. Undergraduates who expressed interest in participating were sent a survey link containing the informed consent form and screening questionnaires. Eligible participants were then sent the baseline questionnaires, and those who completed it were randomised to the active or control intervention group. The first participant was randomised by a coin flip, and participants were alternated to each group thereafter. Each group was sent a video to watch which contained the intervention information within 2 days of randomisation. All the surveys and intervention videos were set up and administered on Qualtrics survey engine. Over the next 14 days, participants in both groups were emailed/messaged daily and asked to report their engagement with the intervention material in the prior 24-hr period. The active intervention group was asked ‘Did you practice the imagery technique yesterday the [date inserted]?’ on a yes/no scale, and ‘if so, how many times?’. The control group was asked ‘Did you think about the health information about binge drinking yesterday the [date inserted]?’ on a yes/no scale, and ‘if so, how many times?’. The average number of times that participants engaged with the intervention (averaged across available email responses) quantified engagement with the intervention. Participants who did not respond to three consecutive, or 7 in total follow-up emails, were withdrawn (see Fig. 1). Two weeks after exposure to the active or control intervention video, participants completed the outcome questionnaires (for comparison with baseline). Participants were debriefed and received £15 upon completion of the study.

### Analytical Plan

As this was an online intervention study, we expected to apply quality control protocols to exclude participants who did not practice the imagery technique over the 2-week follow-up period. Indeed, when we conducted an intention to treat analysis with all participants who completed the intervention and follow-up stages (see Fig. 1: active *n* = 26, control *n* = 29), the intervention effects were marginal. After excluding participants who did not practice the intervention during the 2-week follow-up period (3 participants, active *n* = 24, control *n* = 28), significant group differences emerged. This paper reports this per protocol analysis, i.e. following these exclusions (see Fig. 1).

IBM SPSS Statistics 26 was used for data analysis. Questionnaires were scored in line with the factor (subscale) structure and scoring protocol suggested by the validation studies reported earlier. Each questionnaire scale (total score or subscales) was entered into a mixed ANOVA with the variables group (active, control) as between-subjects factor and timepoint (baseline, follow-up) as within-subjects factor. A significant interaction between group and timepoint would indicate that the interventions differentially changed the outcome measure from baseline to follow-up. A main effect of time would indicate a change in the outcome from baseline to follow-up. A main effect of group with no interaction would suggest a failure to match groups. Significant interactions were followed up with specific ANOVAs testing the timepoint effect in each group and the group effect at each timepoint.

The assumption of normal distribution was violated for some outcome measures. However, ANOVA is regarded as being robust against violations of this assumption [52]. The assumption of equality of variance was met for all outcome measures except for Daily Drinking and Self-efficacy Frequency of Drinking. The outcome measures which showed a significant group by time interaction met the assumptions of normality and homogeneity of variance, indicating that the key findings were not due to violations (see Table 2).

## Results

### Participants and Exclusions

Participants who were included in the final analysis (*N* = 52) versus participants who completed baseline but were subsequently excluded for not engaging with the intervention or assessments (*N* = 24, see Fig. 1) did not differ significantly in age, problematic drinking or drinking to cope with negative affect. However, a greater proportion of men (56.3%) versus women (25.0%) was excluded, *X*^*2*^ (1, *N* = 76) = 5.71, *p* = 0.031, suggesting selective attrition of males. Exclusion rate did not differ between intervention groups, *X*^*2*^ (1, *N* = 76) = 0.97, *p* = 0.460. Baseline characteristics were matched between the active and control group (see Table 1).

**Table 1:** Mean (SD, range) of questionnaire data reported by the active and control intervention group

		Groups		*p*
		Active (*n* = 24)	Control (*n* = 28)	
Age		20.63 (1.84, 18–24)	20.21 (1.62, 18–24)	.395
Gender ratio (M/F)		2/22	5/23	.430
Drinking frequency	Daily	4.17%	7.14%	.782
	Weekly	91.67%	92.86%	
	Monthly	4.17%	0	
AUDIT score		15.88 (6.16, 7–26)	14.71 (4.44, 8–25)	.435
DTCC items endorsed (%)		51.55 (25.74, 17.14–100)	46.84 (19.98, 14.29–88.57)	.461
Email response rate during the follow-up (%)		93.45 (8.40, 71.43–100)	86.48 (16.53, 50–100)	.068
Average number of times of practicing/thinking about the intervention in the previous day		0.78 (0.72, 0.07–2.46)	0.66 (0.44, 0.07–1.75)	.435

### Outcome Measures

**Fig. 2:**
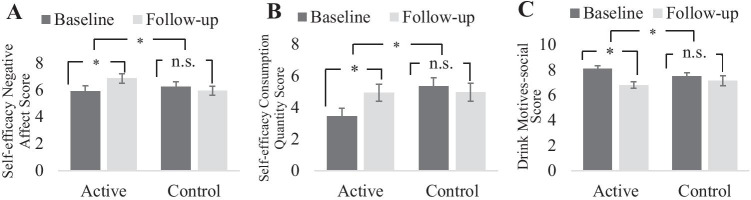
Outcome measures that changed from baseline to follow-up timepoints differentially between the active and control group. The figure highlights three intervention effects: increased self-efficacy of control over alcohol drinking in negative affect situations **A**, increased self-efficacy of control over alcohol consumption quantity **B** and decreased alcohol drinking for social motives **C** from the baseline to follow-up timepoint in the active compared to the control group. Error bars denote standard error of means. *p < .05 for the interaction term and paired contrasts in ANOVA (*F* statistics)

As shown in Table 2 and Fig. 2, a significant interaction between group and timepoint was found for Self-efficacy Negative Affect, *F* (1, 50) = 6.58, *p* = 0.013, η_p_^2^ = 0.116; Self-efficacy Consumption Quantity, *F* (1, 50) = 5.00, *p* = 0.030, η_p_^2^ = 0.091 and Drink Motives-social, *F* (1, 50) = 4.50, *p* = 0.039, η_p_^2^ = 0.083. Specific contrasts showed that for the active intervention group, there was a significant increase from baseline to follow-up for Self-efficacy Negative Affect, *F* (1, 23) = 8.64, *p* = 0.007, η_p_^2^ = 0.273 and Self-efficacy Consumption Quantity, *F* (1, 23) = 6.49, *p* = 0.018, η_p_^2^ = 0.220, and a significant reduction from baseline to follow-up for Drink Motives-social, *F* (1, 23) = 19.79, *p* < 0.001, η_p_^2^ = 0.462. However, for the control intervention group, no significant difference was observed between baseline and follow-up scores for Self-efficacy Negative Affect, *F* (1, 27) = 0.73, *p* = 0.400, η_p_^2^ = 0.026; Self-efficacy Consumption Quantity, *F* (1, 27) = 0.43, *p* = 0.518, η_p_^2^ = 0.016 or Drink Motives-social, *F* (1, 27) = 0.98, *p* = 0.331, η_p_^2^ = 0.035. Group contrasts showed that there was no group difference for Self-efficacy Negative Affect at baseline, *F* (1, 50) = 0.41, *p* = 0.526, η_p_^2^ = 0.008, or at follow-up, *F* (1, 50) = 3.52, *p* = 0.067, η_p_^2^ = 0.066; also no group difference for Drink Motives-social at baseline, *F* (1, 50) = 2.45, *p* = 0.124, η_p_^2^ = 0.047, or at follow-up, *F* (1, 50) = 0.51, *p* = 0.480, η_p_^2^ = 0.010. However, Self-efficacy Consumption Quantity was significantly larger in the control than the active intervention group at baseline, *F* (1, 50) = 6.45, *p* = 0.014, η_p_^2^ = 0.114, but not at follow-up, *F* (1, 50) = 0.001, *p* = 0.973, η_p_^2^ = 0.000. The findings suggest that compared to the control group, the active group showed an increase in self-efficacy of control over negative affect drinking and consumption quantity and a greater decline in the belief that they would drink for social reasons from baseline to follow-up.

**Table 2:** Mean (SD, range) of outcome measures at baseline and two weeks follow-up timepoints for the active (FIT) and control group

	Mean (SD, range)				*p*				
	Active group (*n* = 24)		Control group (*n* = 28)		Main effect of group	Main effect of timepoint		Group × timepoint interaction	
Questionnaire (*α*)	Baseline	Follow-up	Baseline	Follow-up					
Daily drinking (0.66)	64.80 (43.22, 1.10–185.50)	45.36 (34.13, 0.00–139.00)	46.72 (25.00, 7.10–128.20)	29.05 (19.02, 0.00–81.50)	**.022**	**< .001**		.851	
Protective Behaviours (0.73)	3.36 (0.61, 2–4.40)	3.59 (1.10, 1.00–5.40)	3.64 (0.49, 2.85–4.70)	3.71 (1.02, 1.25–6)	.301	.266		.557	
Self-efficacy Negative Affect (0.87)	5.89 (2.08, 2.22–9.56)	6.85 (1.69, 3.56–9.33)	6.24 (1.86, 2.67–10)	5.93 (1.82, 1.00–9.33)	.536	.195		**.013**	
Self-efficacy Frequency of Drinking (0.93)	7.56 (2.92, 1.33–10)	8.06 (2.24, 3.67–10)	8.55 (2.11, 2.67–10)	7.94 (2.78, 2–10)	.440	.900		.197	
Self-efficacy Positive Mood/Social Context (0.68)	4.88 (1.94, 1.83–8.17)	5.56 (1.82, 2.33–8.67)	4.54 (1.71, 1.83–9.17)	4.85 (1.70, 2.33–9.50)	.221	.070		.482	
Self-efficacy Consumption Quantity (0.88)	3.44 (2.58, 0–8)	4.94 (2.68, 0.50–10)	5.35 (2.82, 0–10)	4.96 (2.94, 0–10)	.137	.197		**.030**	
Drink Motives-social (0.75)	8.08 (1.17, 5.60–10)	6.80 (1.24, 4.80–9.20)	7.47 (1.55, 2.60–9.60)	7.14 (2.06, 0.8–9.80)	.730	**.001**		**.039**	
Drink Motives-coping (0.94)	5.41 (2.07, 2.11–10)	4.69 (1.97, 1.49–8.61)	5.10 (2.02, 1.88–8.92)	4.79 (2.30, 1.10–8.67)	.852	**.019**		.337	
Drink Motives-enhancement (0.78)	6.53 (1.70, 3–9.20)	5.83 (1.73, 2.80–9)	6.26(1.76, 1.40–8.40)	6.10 (1.86, 2–9.40)	.995	.058		.228	
Drink Motives-conformity (0.92)	3.54 (2.40, 0–8.20)	2.98 (2.20, 0–8.60)	3.29 (2.71, 0–9)	2.95 (2.54, 0–7)	.817	.146		.715	
Anxiety (0.87)	7.96 (4.55, 1–17)	6.33 (5.37, 0–17)	8.64 (5.63, 0–21)	6.79 (5.88, 0–21)	.688	**.002**		.829	
Depression (0.88)	9.96 (5.15, 2–21)	7.17 (5.36, 1–20)	9.68 (5.54, 1–24)	7.43 (5.56, 0–21)	.995	**< .001**	**.602 **		

The right-hand columns show *p* values from mixed ANOVAs testing the main effects and interaction of group and timepoint for each outcome measure. A significant interaction reveals an intervention effect. Cronbach’s *α* reliability score for each outcome measure is reported in brackets following the name of the measure. Controlled Drinking Self-Efficacy Scale is reported with four subscales: Self-efficacy Negative Affect, Self-efficacy Frequency of Drinking, Self-efficacy Positive Mood/Social Context, and Self-efficacy Consumption Quantity. Drinking Motives Questionnaire — Revised is reported with four subscales: Drink Motives-social, Drink Motives-coping, Drink Motives-enhancement and Drink Motives-conformity

*Anxiety* Generalised Anxiety Disorder Questionnaire, *Depression* Patient Health Questionnaire Depression Scale, *Daily Drinking* Daily Drinking Questionnaire Revised (sum of units consumed over a two-week period time), *Protective Behaviours* Protective Behaviour Strategies Scale — Revised

Unexpectedly, there was a significant main effect of group but no interaction for Daily Drinking, suggesting a failure to match intervention groups for daily drinking quantity. To test the impact of this confound, Daily Drinking score was averaged between baseline and follow-up and entered as a covariate into ANCOVAs, alongside the group and timepoint variables, for each of the three outcomes for which there was a significant interaction. Results showed that the group by timepoint interactions remained significant for Self-efficacy Negative Affect, Self-efficacy Consumption Quantity and Drink Motives-social when average Daily Drinking was controlled. Finally, there was a significant main effect of timepoint for Daily Drinking, Drink Motives-social, Drink Motives-coping, Anxiety and Depression, indicating that these measures decreased from baseline to follow-up in both groups. These effects could be due to the interventions/observation in both groups causing in a reduction in these outcomes, or differences between the two timepoints at which the measures were collected.

## Discussion

As noted in the introduction, drinking to cope with negative affect is a key risk mechanism underpinning problematic drinking in students (and other populations), so mental imagery-based interventions have been devised to mitigate negative affect reactivity. As far as we are aware, the current study was the first to test whether a FIT intervention linking experience of negative affect to retrieval of mental imagery of adaptive strategies would reduce drinking frequency, drinking-related measures (drinking motives, self-efficacy, protective behaviour strategies) and psychiatric symptom severity, in hazardous, student, negative affect drinkers. The study found that compared to the control group, the participants in the active (FIT) intervention group showed greater improvements in three drinking-related measures, although there was no difference in actual drinking frequency. First, the active intervention group showed a significant increase in their perceived self-efficacy to control over drinking triggered by negative affect in the past 2 weeks. The active intervention group endorsed greater confidence that they would not drink heavily in response to such items as: ‘When you are depressed’. Second, the active intervention group showed a significant increase in self-efficacy of control over the quantity of alcohol anticipated in the next 6 months. Specifically, they reported increased confidence that they would not drink heavily in response to such items as: ‘Can you make sure that you do not have more than three drinks on any time that you have a drink’. Finally, the active intervention group showed a significant reduction in social drinking motives across the past 2 weeks. Specifically, they perceived that their drinking was less motived by such items as: ‘To be sociable’. These findings provide initial evidence that 2 weeks of online training to respond to negative affect drinking triggers by retrieving future adaptive strategies can improve drinking-related outcomes, in hazardous, student, negative affect drinkers. The current study added to the existing literature that imagery retrieval training approach has utility for improving substance use outcomes [25, 33, 53].

The current findings extend previous FIT studies which have demonstrated intervention effects on weight loss in obesity [29, 30], reductions in self-harm [31] and increased grit [32]. These results also add to the growing number of studies reporting positive outcomes from online imagery training. This includes three acute online episodic positive future thinking studies showing reduced demand for food and substances [28, 34, 35] and a FIT study using a mixture of face to face and electronic delivery to promote weight loss [30]. The current study is unique in screening a specific cohort of hazardous student negative affect drinkers, delivering personalised online imagery retrieval training, and using electronic reminders to promote the integration of learning into daily life over a 2-week follow-up period. This current pilot design can be used as a model for advancing online FIT delivery, although using pilot trials to justify larger RCTs is complex and needs to be approached carefully [54].

One paradoxical finding in the current study is that the active intervention increased self-efficacy of control over negative affect drinking (Self-efficacy Negative Affect), but did not reduce drinking to cope with negative affect (Drink Motives-coping), despite these two scales measuring similar constructs. A possible explanation lies in the framing of these two questionnaires. The self-efficacy questionnaire asked participants to report their confidence that in the ‘next 6 months’ they would not ‘drink heavily’ in response to negative affect, whereas the drinking motive questionnaires asked participants to report whether in the ‘last 2 weeks’ their drinking had been motivated by negative affect. We might anticipate a bigger intervention effect on perceived control over negative affect drinking in the future compared to recent past if such confidence accrued across the 2-week training period. This possibility might be tested by measuring Drink Motives-coping motives in the past day, for each day of the 2-week training period, in the expectation that coping motives would decline across time. Alternatively, one might accept the null effect on Drink Motives-coping as a true negative and reduce confidence in the intervention effect on Self-efficacy Negative Affect. The fact that the active intervention did not result in any change in Drink Motives-coping is concerning, as it was specifically designed to modify this construct. Future studies should adopt a longer follow-up period with repeated testing to investigate whether Drink Motives-coping incrementally declines with extended practice of the imagery technique.

The active intervention encompassed multiple components, and it is unknown which specific mechanism was responsible for the improvement in the drinking-related outcomes. The most trivial possibility is that the active intervention produced a demand effect wherein participants acquired a sense of what was expected of them by the experimenter and responded accordingly in the outcome measures. This demand interpretation is weakened by the finding that the active intervention only modified some outcome measures and not others (e.g. Self-efficacy Negative Affect but not Drink Motives-coping). This specificity suggests that the active intervention changed a more specific set of beliefs, largely connected to perceived control over drinking, rather than merely engaging a general wish to please the experimenter. Our favoured interpretation is that the active intervention attenuated reactivity to negative affect, i.e., made participants more resilient to negative affect leading them to perceive greater control over drinking. Such growth in resilience to negative affect has been seen with mindfulness-based interventions [13–18] and imagery-based interventions including episodic positive future thinking [20–22] and FIT [31]. As a component of this, FIT might have worked via the establishment of implementation intentions, that is, through the formal rule to respond to negative affect by retrieving an adaptive strategy. Implementation intention studies have demonstrated that setting clear risk-response rules improves behaviour change outcomes [55–60] and can attenuate affective reactivity [61]. However, inconsistent with this claim, the study found no group difference in symptoms of depression or anxiety at follow-up. Such differences would be expected if FIT had improved resilience to negative affect. It is possible that assays of mood (as opposed to psychiatric symptom severity) would have greater sensitivity to this proposed effect of FIT [62, 63] and mediate the effect of FIT on the drinking-related outcomes. Future FIT studies should include assays of mood and affective resilience to test this possibility. The final possibility is that the practice of retrieving adaptive strategies by the FIT group increased engagement with and the value ascribe to alternative rewarding activities, which reduced engagement with alcohol. This claim is supported by the finding that engagement with substance-free alternative activities can reduce alcohol use problems in college students [64] and adolescents [65], although it is not known if this mechanism operated in the current study. As with mood/resilience measures, future FIT studies should include assays to detect changes in engagement with alternative activities to test the role of this candidate-mediating mechanism.

Several limitations of the current study are noteworthy. One limitation is low statistical threshold for significance. The per protocol analysis yielded significant group differences only after three participants who did not practice the intervention at all were excluded. Accordingly, future studies should consider ways to reinforce repeated imagery practice to increase the magnitude of the effects. Another weakness of the current study was the large number of outcome measures which increases the possibility of detecting a group difference due to chance. A future larger-scale trial should use the current pilot results to make stronger a priori judgements about which measures to include and the predicted effects. Perhaps the key limitation of the current study was the lack of group effect on drinking frequency. This may have arisen from the relatively short follow-up period which reduces the likelihood of detecting a change in actual behaviour [38]. A previous study has demonstrated that mindfulness-based intervention showed more benefits on drinking frequency than CBT-based relapse prevention at 12-month follow-up [18], suggesting a longer period might be crucial. Finally, the sample was largely female, so it remains to be seen if the same intervention effects would be obtained in males. It is noteworthy that a previous related study reported effects on reduced drinking frequency and drunkenness only in females [66], so there is a concern that FIT might also have this gender bias. Related to this, the sample size was relatively small, due to inclusion criteria carefully selecting the specific risk population. Previous FIT studies have used sample sizes smaller than 50 and have shown effectiveness in reducing snacking [29] and self-harming [31], so the current study is not inconsistent with these. Therefore, the current proof-of-concept intervention design gives confidence in the general approach, but a future full-sized trial is needed to confirm efficacy.

In conclusion, online training to respond to negative affect drinking triggers by retrieving adaptive strategies increased self-efficacy of control over negative affect drinking and consumption quantity and reduced drinking for social reasons at 2-week follow-up, in hazardous, student, negative affect drinkers. The current study is distinguished from the previous work by selecting hazardous drinkers who drink to cope with negative affect, delivering the intervention entirely online and extending training for a 2-week period, supporting the utility of FIT for substance use outcomes. Although limitations of low statistical threshold, short follow-up period; the small, female dominant sample and absence of potentially more sensitive measures may have masked benefits and limited the generalisability of the intervention effect, the current findings provide initial evidence for the effectiveness of this online intervention package and justify a larger scale trial and more experimental work on imagery-based affect mitigation strategies.
